# Availability of adequately iodized in Northwest Ethiopia: a cross-sectional study

**DOI:** 10.1186/s13690-017-0201-0

**Published:** 2017-07-31

**Authors:** Zegeye Abebe, Amare Tariku, Ejigu Gebeye

**Affiliations:** 1 Department of Human Nutrition, Institute of Public Health, College of Medicine and Health Sciences, University of Gondar, Gondar, Ethiopia; 2 Department of Epidemiology and Biostatistics, Institute of Public Health, College of Medicine and Health Sciences, University of Gondar, Gondar, Ethiopia

**Keywords:** Iodized salt, Knowledge, Residence, Distance, Ethiopia

## Abstract

**Background:**

Universal salt iodization is the most cost-effective, safe and sustainable strategy to eliminate iodine deficiency disorders. However, little is known about the availability of adequately iodized salt in the northwestern part of Ethiopia. Thus, the aim of this study was to assess the availability of adequately iodized salt at the household level and associated factors in Dabat District, northwest Ethiopia.

**Methods:**

A community-based cross-sectional study was conducted from February 21 to March 31, 2016. We included 705 households in the study. A stratified multistage followed by simple random sampling technique was employed to select households. The level of salt iodine content was determined using the rapid field test kit. Accordingly, the value of <15 parts per million (PPM) and ≥15 PPM with the corresponding color chart on the rapid test kit were used to classify the level of iodine content in the sampled salt. A multivariable binary logistic regression model was fitted to identify factors associated with the availability of adequately iodized salt. Adjusted Odds Ratio (AOR) with the corresponding 95% Confidence Interval (CI) was calculated to show the strength of association.

**Results:**

This study indicated that about 33.2% [95% CI: 29.6, 36.7%] of households had adequately iodized salt. Urban residence (AOR = 2.15, 95% CI: 1.23, 3.76), use of packed salt (AOR = 2.23, 95% CI: 1.01, 4.89), and good respondents' knowledge on iodized salt use (AOR = 1.49, 95% CI: 1.08, 2.08) were positively associated with the availability of adequately iodized salt. However, longer distance to buy salt was inversely related with availability of adequately iodized salt (AOR = 0.68, 95% CI: 0.48, 0.99).

**Conclusions:**

The availability of iodized salt is well under the WHO recommendation in Dabat District in spite of the fact that Ethiopia has been implementing universal salt iodization since the last five years. Therefore, intensifying strategies targeting to enhance community awareness on the benefit and handling practice of iodized salt is essential to improve availability of iodized salt. In addition, the focus needs to be on improving accessibility of iodized salt.

## Background

Iodine is important for thyroid hormone production, hence, it has a critical role in supporting brain development, control of metabolic function, and reproduction [1, 2]. According to the World Health Organization (WHO) recommendation, a preschool children needs about 90 μg of iodine per day to prevent iodine deficiency (ID) [1].

Iodine deficiency is a major public health problem, especially in children and pregnant women [3]. It is known to cause mental retardation, goiter, miscarriage, still birth, premature birth, and increase child mortality, collectively called “iodine deficiency disorders (IDD)” [4–6].

Globally, close to 2 billion population is at risk of ID, while one-third live in areas where natural sources of iodine is low [7]. Universal salt iodization (USI) is recommended as the most cost-effective, safe and sustainable strategy to eliminate IDDs [7]. However, only 75% of the household’s worldwide use iodized salt. This USI coverage is considered as a dramatic increment compared to the 1990 report, 10% [8].

In terms of the region, an optimal iodized salt utilization (90%) is reported in East Asia and Pacific countries. In sub-Saharan Africa, 64% of households are using iodized salt nevertheless the level of utilization widely varies from 10 to 90% in different countries. For instance, utilization of iodized salt is less than 10% in Sudan, Mauritania, Guinea-Bissau, and Gambia, whereas, in Burundi, Kenya, Nigeria, Tunisia, Uganda, and Zimbabwe, it is more than 90% [9].

In Ethiopia, utilization of adequately iodized shows a gradual improvement, 4% in 2000 to 15% in 2011 and 23% in 2014 [10, 11]. Disparities in level of utilization are detected with residence and regions. As illustration, iodized salt utilization is high in urban areas and in Amhara, Oromia, and Tigray regions compared to other regions [11].

Furthermore, level of availability of adequately iodized salt depends on other socio-demographic characteristics. Family size [12], economic status [13], mothers educational status [14, 15], knowledge about iodized salt use and prevention of IDDs [14, 16], and salt handling practices [17–20] are significantly associated with availability of adequately iodized salt.

Ethiopia is a salt producing state, endorsed mandatory salt iodization program and is working with partners, United Nations Children’s Fund (UNICEF) and Micronutrient Initiatives, to reach utilization of iodized salt >90% thereby to mitigate ID [21]. But, still only 23.2% of the households use adequately iodized salt [11]. In addition, ID remains the major public health problem [22, 23] among school children and pregnant women [20, 24]. Even there is high prevalence of goiter in Dabat District, the study area [25]. However, there is a limited information on availability of universal iodized salt in Dabat District. Therefore, this study aimed to investigate the household availability of adequately iodized salt and associated factors in Dabat District, northwest Ethiopia.

## Methods

### Study design and setting

A community-based cross-sectional study was conducted from February 21 to March 31, 2016, in Dabat District, northwest Ethiopia. The district is found 821 km from Addis Ababa, the capital city of Ethiopia. It lies at an altitude of 1000–2500 meters and has a total population of 175,737 in 26 rural and 4 urban Kebeles (the *smallest administration unit in Ethiopia*). Cereals, such as maize, sorghum, wheat, and barley are the main staple crops cultivated in the district. The Health and Demographic Surveillance System (HDSS) site was also located in Dabat District. The HDSS site has been running since 1996 and hosted by the University of Gondar. The surveillance site covers 13 randomly selected kebeles (4 urban and 9 rural kebeles) in different ecological zones (high land, middle land, and lowland).

### Sample size and sampling procedure

The sample size of the study was calculated using Open Epi version 2.3 software with the following assumptions; 33% as the prevalence of availability of iodized salt at the household level [14], 95% Confidence level, and a 5% margin of error. Finally, the sample size of 714 households was obtained by considering 5% non-response rate and a design effect of 2. A multistage stratified sampling followed by simple random sampling technique was employed to reach the households. Initially, households were stratified into urban and rural kebeles. Of the total13 kebles in the HDSS site, 4(1 urban and 3 rural kebeles) were selected by lottery method. A total number of households in the selected kebeles were obtained from HDSS site, and then the total number of households included in the study was proportionate-to-the household size. Finally, a simple random sampling technique was employed to select the households. Women who were majorly involved in food preparation of the household were interviewed.

### Data collection instrument and procedure

We used a structured interviewer-administered questionnaire to collect data. The questionnaire was first prepared in English and was translated into the local language (Amharic) and finally, back translated to English to maintain consistency.

The pretest was done on five percent of the sample out of the study area. Two days training was given for data collectors and supervisors. A total of seven data collectors (an environmental health professional, and six permanent data collectors of the HDSS site) and two supervisors (two public health experts) were recruited for the study. The rapid test of salt iodine content was done by an environmental health professional who had adequate training (certificate of Training of Trainers) in the determination of salt iodine content. Daily supervision and feedback were carried out by the investigators and supervisors during the entire data collection period to maintain the quality of the data.

### Salt iodine content determination

A tablespoon of salt was collected from each household, and the MBI international Rapid Test Kit (RTK) was used to determine the level of salt iodine content. The small cup in the kit was filled with salt, and made the cup surface flat. Two drops of test solution from white ampoule were added to the surface of the salt by piercing the white ampoule with a pin and gently squeezing the ampoule. The iodine content in the salt was determined within one minute by comparing the color change on the salt with the color chart. The value 0 parts per million (PPM), <15 PPM and ≥15 PPM with the corresponding color chart on the rapid test kit were used to classify the level of iodine in the sampled salt. If no color appears (after 1 min), 5 drops of the recheck solution from red ampoule was added to a fresh salt sample and followed by 2 drops of test solution on the same salt sample [1, 11]. Then, a comparison was done with the color chart indicators for salt iodine content. Finally, Availability of adequately iodized salt was considered when the household sampled salt had ≥ 15 ppm iodized salt [1], otherwise, it was classified to inadequately iodized salt.

### Assessment of wealth status and maternal knowledge

The household’s wealth index was determined using Principal Component Analysis (PCA) by considering the household assets, quantity of cereal products, type of house, livestock and agricultural land ownership. First, variables were coded between 0 and 1. Secondly, variables entered and analyzed using PCA, and those variables having a communality value of greater than 0.5 were used to produce factor scores. Finally, the factor scores were summed and ranked into tertiles as poor, medium and rich.

Similarly, the knowledge of the respondents towards iodized salt use were computed by using nine knowledge item questions, including the importance of iodized salt, disorders resulted from ID, food sources of iodine, appropriate place of salt storage, time to add salt during food preparation, salt storage material, and existence of law prohibiting selling non-iodized salt in Ethiopia. Accordingly, the PCA was also used rank respondents' knowledge into not knowledgeable and knowledgeable.

### Data processing and analysis

Data were checked and entered into EPI-Info version 7 and analyzed using SPSS version 20 statistical software. The availability of adequately iodized salt, the outcome variable, was dichotomized into inadequately iodized (0-14PPM) and adequately (≥15PPM) iodized salt based on the result of rapid test. Descriptive statistics were used to summarize variables. A binary logistic regression was fitted to identify factors associated with the availability of adequately iodized salt. Variables with a p-value less than <0.2 in the bivariable analysis and those variables which frequently showed significant association with the availability of adequately iodized salt in the previous studies, regardless of the p-value in the current study, were also fitted into the multivariable binary logistic regression analysis. A backward likelihood ratio method was employed. Both Crude Odds Ratio (COR) and Adjusted Odds Ratio (AOR) with the corresponding 95% Confidence Interval were estimated to show the strength of association. In multivariable analysis, variables with a *p*-value of <0.05 were considered as statistically significant.

## Results

### Socio-demographic and economic characteristics

A total of 705 households were included in the study, which makes a response rate of 98.7%. The median age of the respondents was 35 years with Inter-quartile Range (IQR) of ±10 years. About 434 (61.6%) of the households had a family size of greater than five. Three-quarters of the respondents, 539 (76.5%), were illiterate (Table 1).

**Table 1:** Socio-demographic and economic characteristics of respondents, Dabat District, northwest Ethiopia, March 2016 (*n* = 705)

Variables	Frequency	Percentage
Age of respondents		
16–24	36	5.1
25–34	268	38.0
35–49	330	46.8
50–79	71	10.1
Residence		
Urban	151	21.4
Rural	554	78.6
Mother’s marital status		
Currently married	616	87.4
Currently unmarried^c^	99	12.6
Religion		
Orthodox	689	97.7
Muslim	16	2.3
Mother’s education		
Illiterate	539	76.5
Primary	97	13.8
Secondary and above	69	9.8
Husband’s education		
Illiterate	390	55.3
Primary	225	31.9
Secondary and above	90	12.8
Mother’s occupation		
Housewife	309	43.8
Farmer	319	45.2
Merchant	32	4.5
Government employee	25	3.5
Others^b^	20	2.8
Husband’s Occupation		
Farmer	571	81.0
Merchant	36	5.1
Government employee	72	10.2
Others^a^	26	3.7
Family size		
<6	271	38.4
≥6	434	61.6
Wealth status		
Poor	237	33.6
Medium	233	33.0
Rich	235	33.3

^a^daily laborer, pension; ^b^students, daily laborer; ^c^single, divorced, widowed

### Availability of adequately iodized salt

In Dabat District availability of iodized salt was 33.2% (95% CI: 0.296, 0.367). About 675 (95.7%) of households used the unpacked type of salt for food preparation out of which 457 (67.7%) were inadequately iodized. A substantial proportion (95 and 91%, respectively) of salt was covered and stored away from a fire. However, 355 (50.4%) of the salt was added at the beginning and middle of food preparation (Table 2).

**Table 2:** Availability of adequately iodized salt and handling practices, Dabat District, northwest, Ethiopia, March 2016 (*n* = 705)

Variables	Frequency	Percentage
Type of salt		
Packed	30	4.3
Unpacked	675	95.7
Addition of salt during food preparation		
At the beginning and the middle	355	50.4
At the end	350	49.6
Salt exposure to sunlight		
Yes	35	5.0
No	670	95.0
Washing of salt to remove impurities		
Yes	13	1.8
No	692	98.2
Quantity of salt purchased commonly		
<1 kg		
1 kg	439	62.3
2–5 kg	153	21.7
>5 kg	113	16.0
Place of salt storage		
Near to the fire	65	9.2
Away from the fire	640	90.8
Salt storage material		
With closed container	669	94.9
Without closed container	36	5.1
Duration of household salt storage		
1-8 weeks	642	91.1
≥9 weeks	63	8.9
Salt iodine content		
0-14PPM	472	66.8
≥15 PPM	234	33.2
Distance travel to buy iodized salt		
≤60 min walking	509	72.2
>60 min walking	196	27.8

### Respondent’s knowledge and attitude towards iodized salt use

Nearly half, 320 (45.4%), of the respondents had good knowledge while two-third, 441 (62.6%), of the respondents had favorable attitude towards iodized salt use. Nevertheless, nearly half, 314 (44.5%), of them didn’t know the importance of using iodized salt (Table 3).

**Table 3:** Respondent’s knowledge and attitude towards iodized salt use, Dabat District northwest Ethiopia, 2016 (*n* = 735)

Variables	Frequency	Percentage
Respondent’s knowledge		
Poor	385	54.6
Good	320	45.4
Respondent’s attitude		
Unfavorable	264	37.4
Favorable	441	62.6
Importance of iodized salt^a^		
Prevention of goiter	110	15.6
Growth and development	16	2.3
For health	364	51.6
I don’t know	314	44.5
The richest source of iodine^a^		
Egg	25	3.5
Meat	37	5.2
Milk and milk products	37	5.2
Iodized salt	61	8.7
Fish	8	1.1
Fruit and vegetables	7	1
I don’t know	591	83.8
Disorders of lack of iodine^a^		
Mental retardation	25	3.5
Goiter	174	24.7
Retarded growth	7	1
Abortion	9	1.3
Child mortality	1	0.1
I don’t know	501	71.1
All salts contain iodine		
Yes	73	10.4
No	212	30.1
I don’t know	420	59.6
Existence of law that prevents selling none iodized salt for human/animal consumption		
Yes	44	6.2
No	181	25.7
I don’t know	480	68.1
Test of iodized salt is different from unionized one		
Yes	175	24.8
No	267	37.9
I don’t know	263	37.3
Iodized salt has a harmful effect on health		
Yes	33	4.7
No	590	83.7
I don’t know	82	11.6
Salt obtained from the sea already contain iodine in the right quantities		
Yes	97	13.8
No	386	54.8
I don’t know	222	31.5

^a^multiple responses

### Factors associated with the availability of adequately iodized salt

To assess the effect of selected variables on the availability of iodized salt in the household, both bivariable and multivariable binary logistic regression analyses were fitted. Accordingly, residence, type of salt used, maternal knowledge and distance traveled to buy salt were associated with the availability of iodized salt in the final model. The higher odds of availability of adequately iodized salt were observed among households located in urban kebeles (AOR = 2.15; 1.23, 3.76)) and used packed type of salt (AOR = 2.23; 1.01, 4.89). Similarly, the odds of having adequately iodized salt were 1.49 times (AOR = 1.49; 1.08, 2.08) higher among respondents who had good knowledge towards iodized salt use as compared to their counterparts. Whereas lower odds of availability of adequately iodized salt were found among respondents who traveled a longer distance to buy salt (AOR = 0.68, 95% CI: 0.48, 0.99) (Table 4).

**Table 4:** Factors associated with the availability of adequately iodized salt in the household, Dabat District, northwest, Ethiopia, March 2016 (*n* = 705)

Variables	Availability of iodized salt		Crude Odds Ratio 95% C1	Adjusted Odds Ratio 95% CI
	≥15PPM	<15PPM		
Respondents age in years				
16–24	15	21	1.82 (0.79, 4.22)	*
25–34	84	184	1.16 (0.65, 2.07)	*
35–49	115	215	1.36 (0.78, 2.40)	*
50–79	20	51	1.00	*
Residence				
Urban	63	88	1.60 (1.11, 2.32)	2.15 (1.23, 3.76)
Rural	171	383	1.00	1.00
Mother’s education				
Illiterate	171	368	1.00	*
Primary education	38	59	1.39 (0.89, 2.17)	*
Secondary and above	25	44	1.22 (0.73, 2.06)	*
Husband’s education				
Illiterate	126	264	1.00	*
Primary education	73	152	1.01 (0.71, 1.43)	*
Secondary and above	35	55	1.33 (0.83, 2.14)	*
Mother’s occupation				
Housewife	99	210	1.00	*
Farmer	109	210	1.10 (0.80, 1.54)	*
Merchant	11	21	1.11 (0.52, 2.39)	*
Government employee	9	16	1.19 (0.51, 2.79)	*
Others	6	14	0.91 (0.34, 2.44)	*
Husband’s occupation				
Farmer	13	23	1.00	*
Merchant	185	386	0.85 (0.42, 1.71)	*
Government employee	24	48	0.89 (0.38, 2.05)	*
Others	12	14	1.52 (0.54, 4.24)	*
Family size				
≤ 5	96	175	1.18 (0.85, 1.62)	*
≥ 6	138	296	1.00	*
Household wealth index				
Poor	81	156	1.00	*
Medium	69	164	0.81 (0.55, 1.19)	*
Rich	84	151	1.07 (0.73, 1.56)	*
Type of salt				
Packed	16	14	2.39 (1.15, 4.99)	2.23 (1.01, 4.89)
Non-packed	218	457	1.00	1.00
Salt exposure to sunlight				
No	225	445	1.00	*
Yes	9	26	0.69 (0.32, 1.49)	*
Place of salt storage				
Near to fire	21	44	0.96 (0.56, 1.65)	*
Away from fire	213	427	1.00	*
Salt storage				
With closed material	222	447	1.00	*
Without closed material	12	24	1.01 (0.49, 2.05)	*
Duration of salt storage				
1–8 weeks	212	430	1.00	*
≥9 weeks	22	41	1.09 (0.63, 1.87)	*
Mother’s knowledge				
Poor	113	272	1.00	1.00
Good	121	199	1.46 (1.07, 2.00)	1.49 (1.08, 2.08)
Mother’s attitude				
Unfavorable	81	183	1.00	*
Favorable	153	288	1.20 (0.86, 1.66)	*
Distance travel to buy salt				
≤60 min walking	180	329	1.00	1.00
>60 min walking	54	142	0.69 (0.48, 0.99)	0.68 (0.48, 0.99)

*didn’t appear in the final model (not significant) using Backward LR

## Discussion

It is clear that the use of non-iodized salt by households is the major obstacle in the control of IDDs in a given community [26, 27]. Therefore, regular monitoring of availability of adequately iodized salt is an essential step to eliminate the problem [28, 29]. Cognizant of the fact, the WHO recommendation recommend utilization of the household iodized salt to be greater than 90% to eliminate IDDs [1]. Nevertheless, this study found that only 33.2% (95% CI: 29.6, 36.7%), of households had adequately iodized salt. The result is considerably lower than the national goal of reaching >90%, which alerts the public authorities to consider regular monitoring of the quality of salt at the site of production, wholesale and household level. Strengthening media promotion on efficient utilization of iodized salt, at household level, is very also crucial [30].

On the other hand, the availability of adequately iodized salt in Dabat District (33.2%) is higher than other urban district of Ethiopia: Assosa (26.1%) and Gondar (28.9%) [31, 32]. Likewise, the finding is higher compared to a study in Kenya (26.2%) [33]. This is probably due to improved effort of local government to ensure market availability of iodized salt, the best strategies to address ID in areas where the natural source of iodine is low. The officials are also giving special emphasis in promoting the health benefit of iodized salt through the local medias to bring the desired public awareness [34].

However, availability of adequately iodized salt is significantly lower than the one observed in India (81.9%) [35], and Ethiopia (62.9% [36]. Mother’s poor knowledge towards iodized salt and using unpacked type of salt in 80% of the households could explain the lower prevalence of availability of adequately iodized salt in this study area compared to latter local report. In addition, shorter duration of time in implementation of universal salt iodization compared to India might explain the observed discrepancy.

In this study, respondents from urban kebeles were more likely to have adequately iodized salt compared to those living in the rural settings. This finding is supported by another study in Ethiopia [37]. This could related to better literacy rate and access to media among urban dwellers which might enhance their level of understanding regarding the importance of  iodized salt and information access, respectively. In addition, urban respondents have access to media, and iodized salt everywhere and every time. Furthermore, rural residents may prefer using non-iodized salt because they perceived that its low cost and more potency in test compared to iodized one [38].

The study also detected that the odds of availability of adequately iodized salt were higher in households using a packed type of salt than their counterparts using unpacked type of salt. This report is in agreement with another former study from West Ethiopia [37]. It is documented that loss of iodine in salt from a good quality polyethylene packing material is less than 10% over 18 months period, regardless of the climatic conditions or fine and coarse texture of salt [1]. Given that loss of iodine is common in the case of the unpacked type of salt because of exposure to heat, moisture, and humidity [33, 38].

On the other hand, traveling longer distance to access shops enforces people to purchase a large amount of iodized salt at once which will increase the chance of losing iodine because of storing the salt for a longer period of time [13]. I line to this evidence, the current study indicated that the likelihood of having adequately iodized salt was lower in the respondents who traveled a longer distance to buy iodized salt compared to their counter parts.

Obviously, boosting mother’s knowledge on iodized salt is an important step to ensure appropriate utilization of iodized salt in the household level [14, 28, 32]. Similarly, this study showed that the odds of having adequately iodized salt were higher among respondents who had good knowledge towards iodized salt compared to those who had poor knowledge. This could be related to the positive effect of mother’s good knowledge in appropriately using and storing of iodized salt. A previous study also documented that poor iodine knowledge is the risk factors for IDDs [39].

Adequate training was given to field assistances (data collectors and supervisors) to improve the quality of data. Though mothers were clearly informed about the objective of the investigation, the study is not free from social desirability bias in responding the type of salt use and handling practice. Moreover, the semi-quantitative method was used to measure salt iodine content which may not reflect the actual availability of adequately iodized salt in the study area.

## Conclusions

Despite Ethiopia has been implementing universal salt iodization since the last five years, the availability of iodized salt is well under the WHO recommendation in Dabat District. Therefore, strengthening measures to enhance community awareness on the benefit and handling practice of iodized salt is essential to improve availability of iodized salt. In addition to this, the focus needs to be on improving accessibility of iodized salt. Finally, the authors would like to recommend the future investigators to use iodometric titration method

## Abbreviations

EPHI: Ethiopia Public Health Institute
HDSS: Health, and demographic surveillance system
ID: Iodine deficiency
IDD: Iodine deficiency disorder
PCA: Principal component analysis
PPM: Parts per million
UNICEF: United Nations Children’s Fund
WHO: World Health Organization
